# Influence of major *BCR-ABL1* transcript subtype on outcome in patients with chronic myeloid leukemia in chronic phase treated frontline with nilotinib

**DOI:** 10.18632/oncotarget.27652

**Published:** 2020-06-30

**Authors:** Alexis Genthon, Franck Emmanuel Nicolini, Françoise Huguet, Carole Colin-Gil, Marc Berger, Sandrine Saugues, Alexandre Janel, Sandrine Hayette, Pascale Cohny-Makhoul, Nadine Cadoux, Jean-Michel Cayuela, Lydia Campos, Denis Guyotat, Pascale Flandrin-Gresta

**Affiliations:** ^1^Service d'Hématologie et Thérapie Cellulaire, Institut Lucien Neuwirth, Saint-Priest-en-Jarez, France; ^2^Hematology Department, Centre Leon Berard, Lyon, France; ^3^French Group of CML, Bordeaux, France; ^4^Service d'Hématologie, Institut Universitaire du Cancer de Toulouse Oncopole, CHU de Toulouse, Toulouse, France; ^5^Service d’Hématologie Biologique, CHU Estaing and Université Clermont Auvergne, Clermont-Ferrand, France; ^6^Laboratoire d'Hématologie, Centre Hospitalier Lyon Sud, Pierre-Bénite, France; ^7^Hématologie Clinique, CH Annecy-Genevois, Epagny Metz-Tessy, France; ^8^Laboratoire de Biologie Moléculaire, Hôpital Saint-Louis, Paris, France; ^9^Laboratoire d'Hématologie, CHU de Saint-Etienne, Saint-Etienne, France

**Keywords:** CML, nilotinib, e13a2, e14a2, BCR-ABL1

## Abstract

Chronic myeloid leukemia (CML) is a myeloproliferative neoplasm characterized by the presence of *BCR-ABL1* transcript as a result of reciprocal translocation between chromosome 9 and 22. The most common transcripts subtypes are e13a2 (b2a2) and e14a2 (b3a2). The prognostic impact of the type of *BCR-ABL1* transcript has been the subject of controversies over time. In the imatinib era, several studies have suggested a deeper and faster response in patients expressing e14a2. However, the impact on response after first line therapy with a second-generation tyrosine kinase inhibitor, nilotinib, is unknown.

We retrospectively evaluated 118 patients newly diagnosed with chronic phase CML and treated frontline with nilotinib inside or outside clinical trial in five French centers. Only patients expressing e14a2 or e13a2 transcripts alone were analyzed.

At baseline, 55.3% expressed e14a2, 44.7% expressed e13a2. The median age was 51 years and median follow-up was 49 months. Relative risks of CML at diagnosis were similar according to the ELTS score (*p* = .87). Complete hematological response and complete cytogenetic response rates were similar among groups. Patients expressing e14a2 transcripts compared to e13a2 transcripts had deeper and faster molecular responses, when considering MMR (100% vs 84.1%, *p* = .007) with a median time of 6.7 and 17.1 months or MR^4.5^ (100% vs 59.9%, *p* = .005) with a median time of 39.7 and 70.9 months, respectively. A sustained treatment free remission was observed in 10/10 patients with e14a2 versus 1/3 with e13a2 transcript (*p* = .04).

In conclusion, even treated with nilotinib first line, patients with chronic phase CML expressing *BCR-ABL1* e13a2 transcript have a lower rate of deep molecular responses.

## INTRODUCTION

Chronic myeloid leukemia (CML) is a myeloproliferative neoplasia characterized in chronic phase by increases in myeloid and platelets cells in the peripheral blood and myeloid hyperplasia in the bone marrow [1, 2].

The pathophysiology of CML has been well established since the description of the Philadelphia chromosome (or Ph^1^) by Nowell and Hungerford in 1960 [3]. The disease is characterized by a translocation [4] that consists of a juxtaposition of the *ABL1* gene from chromosome 9 and the *BCR* gene from chromosome 22, coding for a protein with constitutive tyrosine kinase activity, able to be targeted by tyrosine kinase inhibitors [5, 6].

Depending on the site of the breakpoint in the *BCR* gene, the fusion protein can vary in size from 185 kDa to 230 kDa. The break most commonly occurs between exon 13 (e13) and exon 14 (e14 formerly known as b2) or between e14 and exon 15 (e14, formerly known as b3) in a region of approximately 5.8 kb called the major breakpoint cluster or M-BCR. The breakpoint in the *ABL1* gene is generally located between exons a1 and a2.

Therefore, most of the patients with chronic phase (CP)-CML express a 210-kDa BCR-ABL1 (p210^BCR-ABL1^) coded by e13a2 or e14a2 *BCR-ABL1* transcripts. In some cases, both transcripts can be co-expressed [7, 8]. These two different transcripts differ by a 25-extra aminoacids insertion coded by the e14 (b3) exon. A recent study conducted in Europe on more than 45000 CML cases showed a proportion of 39% of e13a2, 62% of e14a2 and e14a2/e13a2 transcript, and this distribution differed by gender and by age [8].

The prognostic impact of the type of *BCR-ABL1* transcript has been the subject of controversies over time. However, in the imatinib era, several studies on a limited number of patients have suggested a deeper and faster response in patients expressing e14a2 [9–14].

Nilotinib (Tasigna^®^; Novartis, East Hanover, NJ, USA) is a BCR–ABL1 inhibitor designed to be more potent and selective than imatinib [15] and shows better efficacy than imatinib for the treatment of patients with newly diagnosed CML in chronic phase [16]. To our knowledge, there are few published data analyzing the influence of transcripts type in patients treated by nilotinib frontline.

The aim of this study was to evaluate the prognostic impact of transcripts type e14a2 or e13a2 in patients with chronic phase CP-CML treated frontline with nilotinib.

## RESULTS

### PCR efficiency

**Figure 1:**
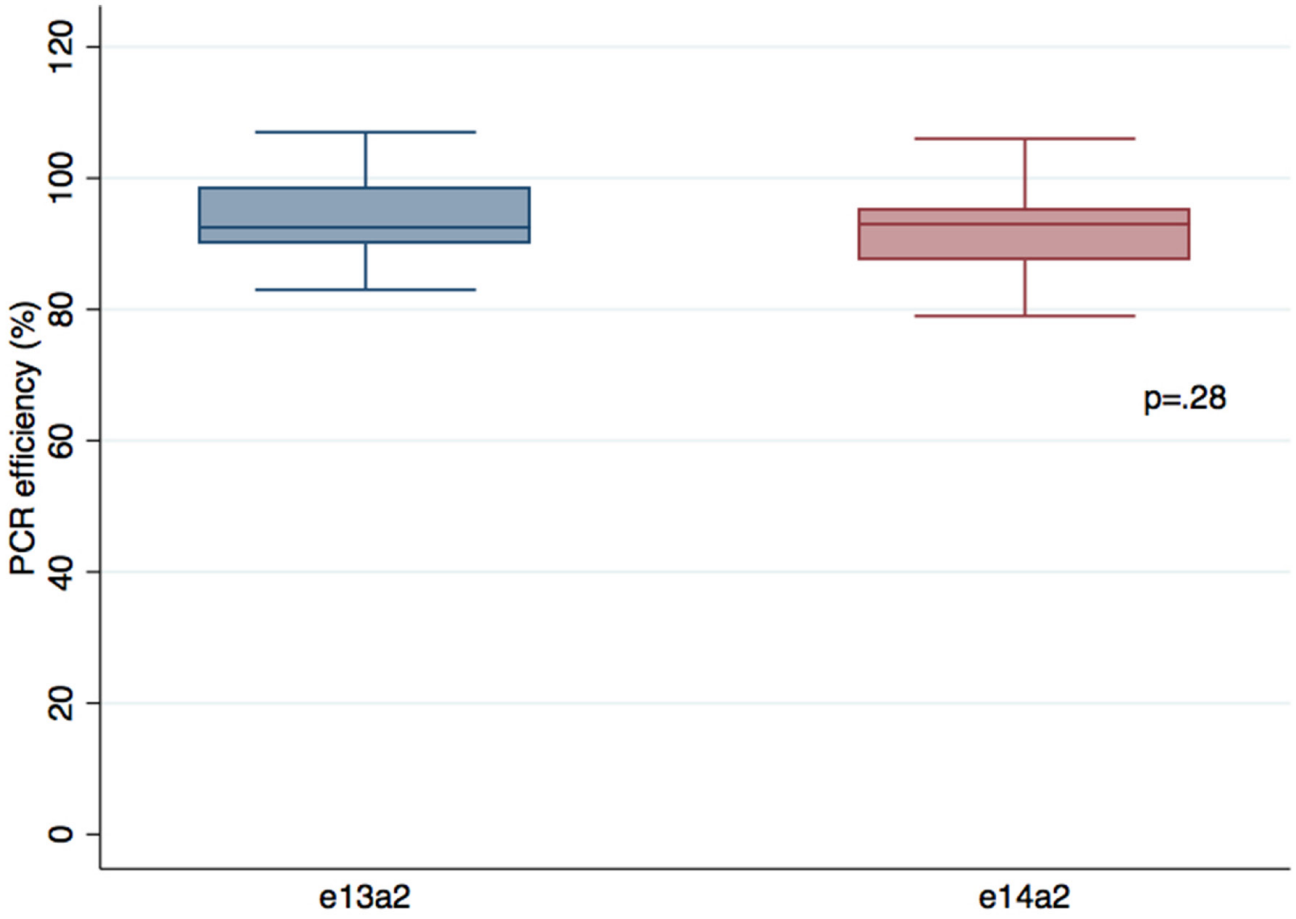
Comparison of PCR efficiencies between e13a2 and e14a2 transcripts. Serial 10 fold dilutions of two different cell lines expressing e13a2 or e14a2 transcripts were performed in 4 distinct experiments (*n* = 48). Results were compared using the Wilcoxon-Mann-Whitney test.

As shown in Figure 1, no differences in terms of PCR efficiencies were observed between the two transcripts, avoiding a bias of amplification that could explain the difference in transcript quantification (*p* = .32). Furthermore, PCR efficiencies were the not different (*p = .34*) when we used a specific e14a2 primer (slope = –3,49; efficiency 93%) or the EAC primers (slope = –3,46; efficiency = 94%) on a e14a2 cell line, suggesting that amplification is comparable between the two transcripts with EAC protocol.

### Patients and disease characteristics

Overall, 118 patients treated frontline with nilotinib for CP-CML were included in this study. Four patients were excluded from this analysis: 3 were treated by interferon (*n* = 2) or by cytarabine (*n* = 1) and 1 patient was in accelerated phase. All analyses were made on the remaining 114 patients. The baseline characteristics of the patients are summarized in Table 1.

**Table 1:** Clinical and Biological Characteristics of patients at diagnosis according to transcript subtype (e13a2 versus e14a2) (n = 114)

Characteristic	*N* = (%) or median [range]		
	e14a2	e13a2	*p* =
*N*	63 (55.3)	51 (44.7)	
Gender, male	33 (52.4)	27 (52.9)	.95
Age (years)	49 [19–84]	53 [18–78]	.27
Hemoglobin (g/L)	118 [69–157]	114 [79–156]	.48
WBC count (10^3^/μL)	106 [4.5–623]	156 [17.6–552.5]	.17
PLT count (10^3^/μL)	405 [83–1999]	311 [104–1315]	.14
Peripheral blasts (%)	1.0 [0–13]	1.1 [0–7]	.98
**ELTS score**			
* Low*	32 (52.5)	24 (48)	.87
* Intermediate*	21 (34.4)	18 (36)	
* High*	8 (13.1)	8 (16)	
**EUTOS score**			
* Low*	55 (90.2)	45 (90)	.98
* High*	6 (9.8)	5 (10)	
**Sokal score**			
* Low*	21 (34.4)	16 (32)	.84
* Intermediate*	27 (44.3)	21 (42)	
* High*	13 (21.3)	13 (26)	
**Hasford score**			
* Low*	24 (39.3)	13 (26)	.08
* Intermediate*	25 (41)	31 (62)	
* High*	12 (19.7)	6 (12)	
**ACA Ph+ or variant translocation**	8 (13.1)	10 (20)	.35
**Median duration of treatment (months)**	42 [3–111]	49 [2–104]	.47
**Median follow-up (months)**	46 [3–121]	53 [3–111]	.97

Abbreviations: ACA Ph+, additional chromosome abnormalities in Ph+ cells; EUTOS, European Treatment and Outcome Study; ELTS, EUTOS long-term survival; PLT, platelets; WBC, white blood cells.

Sixty-three patients (55.3%) expressed e14a2, and 51 patients (44.7%) expressed e13a2 transcripts. The median age at diagnosis was 51 years (range: 18-84 years) for the entire cohort and 52.6% of the patients were male with no significant differences (*p* = .27 and *p* = .95, respectively).

At the time of diagnosis, patients expressing e14a2 had a median white blood cell (WBC) count of 106 G/L compared to 156 G/L in the e13a2 group. Median platelet count was 405 G/L in the e14a2 group and 311 G/L in the e13a2 group. These differences were not significant (*p* = .17, *p* = .14, respectively) between the 2 subgroups.

The patient distribution according to ELTS [17], EUTOS [18], Hasford [19] and Sokal [20] scores was comparable between groups.

There was a similar rate of additional clonal abnormality (ACA) and variant translocation (*p* = .35).

Median follow-up was 49 months for the entire cohort (range: 3–121 months). The median duration of treatment with nilotinib was 42 months (range: 3–111 months) for e14a2 patients and 49 months (range: 2–110 months) for e13a2 patients (*p* = .47). At diagnosis, all patients received 600 mg of nilotinib per day (2 × 300 mg), and during follow up 6 patients (4 in e13a2 subgroup and 2 in e14a2) needed a dose reduction to 450 mg/day for drug toxicity.

### Response to treatment

According to the absence of a systematic difference in prognostic scores, the transcript groups were compared without stratification for those covariates. Only patients with data available at the time of assessment were included.

Response rates for endpoints considered as optimal by the ELN are summarized in Table 2. CHR and CCyR rates were similar in both groups (*p* = .57 and *p* = .52, respectively). There were no differences when considering the EMR between groups, with rates as follows: e14a2 (85.5%) and e13a2 (88%) (*p* = .46).

**Table 2:** Hematological, Cytogenetic and molecular responses at 3, 6, and 12 months follow-up for patients according to transcript type (e13a2 versus e14a2) and ELN endpoints

	CHR at 3 months		CCyR at 6 months		MMR at 12 months	
	e14a2	e13a2	e14a2	e13a2	e14a2	e13a2
*N*	58/62	48/50	54/58	43/48	42/63	26/51
%	93.5	96	93.1	89.6	66.7	50.1
*p* =	.57		.52		.048	

Abbreviations: CHR, complete hematological response; CCyR, complete cytogenetic response; MMR, major molecular response. Results were compared using Student test.

Patients expressing e13a2 had a significantly lower rate of MMR at 12 months (50.1%) compared to those expressing e14a2 (66.7%) (*p* = .048).

**Figure 2:**
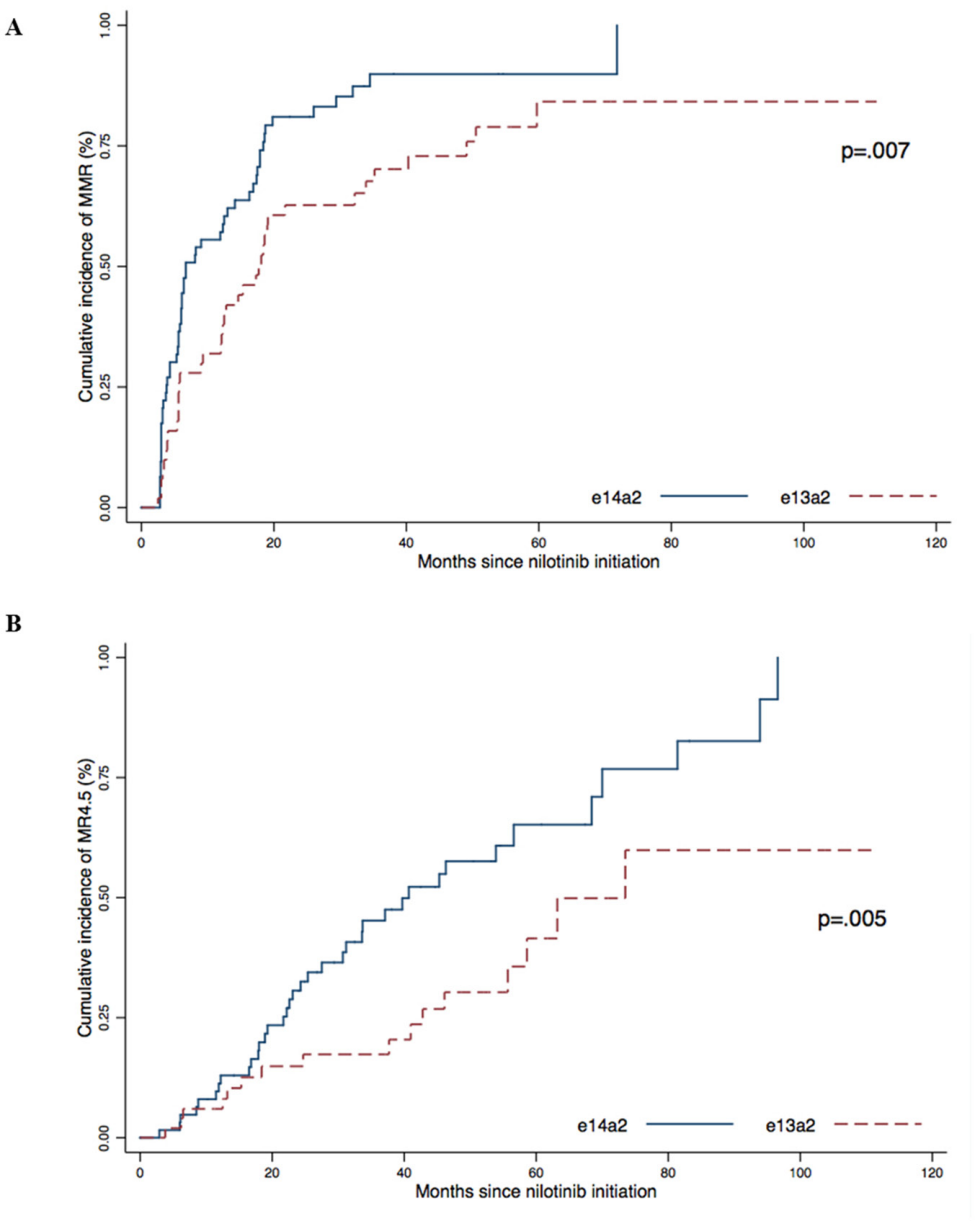
Cumulative incidence of MMR (**A**) and MR4.5 (**B**) according to transcript type (e13a2 or e14a2) were obtained using the Kaplan–Meier method, results were compared using the log-Rank test.

We then analyzed the cumulative incidence of response according to the type of transcript (Figure 2). The cumulative incidence of MMR was 100% in the e14a2 group compared to 84.1% for the e13a2 group (*p* = .007) with a median time of 6.7 months and 17.1 months, respectively.

The cumulative incidence of MR^4.5^ was also higher in the e14a2 group (100%) than in the e13a2 group (59.9%) (*p* = .005) with a median time to MR^4.5^ of 39.7 months and 70.9 months, respectively.

One patient was lost during follow-up and 2 patients progressed: accelerated phase (*n* = 1) and a transformation in Ph^+^ ALL (*n* = 1). These patients underwent allogenic stem cell transplantation. Two patients (1.6%) died during the follow-up: one from a transformation in Ph^+^ acute lymphoid leukemia (ALL) and one from uterine cancer.

**Figure 3:**
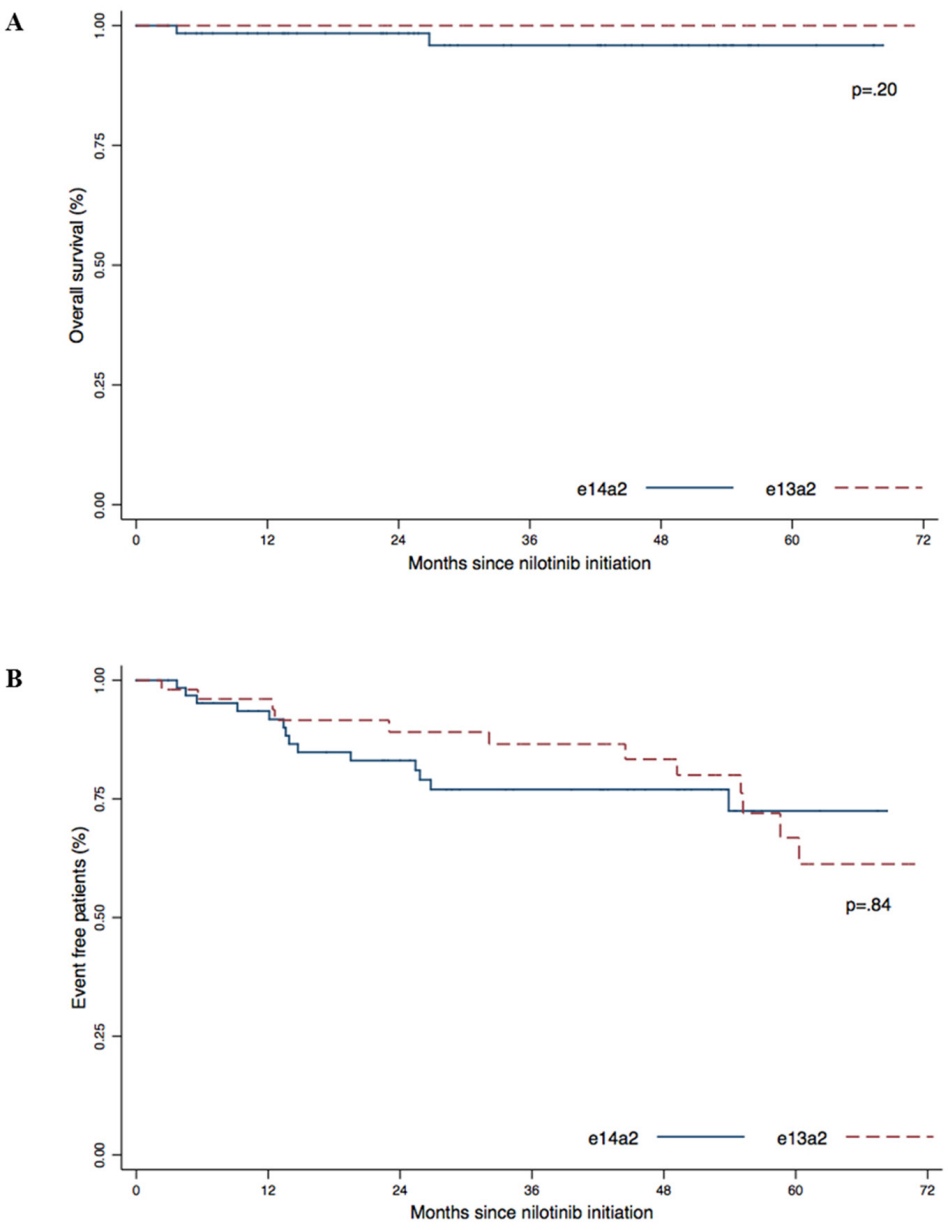
Overall survival (**A**) and Event Free Survival (**B**) according to transcript type (e13a2 versus e14a2). *P*-values were obtained using the log-Rank test.

The estimated 5-year overall survival (OS) was 95.6% for the e14a2 group and 100% for the e13a2 group (Figure 3A). The 5-year event-free survival for the e14a2 group was 72.5% and 66.8% for the e13a2 group (Figure 3B). We did not observed differences in OS (*p* = .20) or EFS (*p* = .84) according to transcript type in our cohort.

### TKI discontinuation

Thirty-nine patients (34.2%) discontinued nilotinib during the follow-up and there was no difference between groups (*p* = .19) with 23 patients over 63 (36.5%) in the e14a2 group and 16 patients over 51 (31.4%) in the e13a2 group. The reasons to stop therapy were failure for 28.2% (*n* = 11), treatment side effects for 35.9% (*n* = 14), an attempt of treatment free remission (TFR) due to sustained deep molecular response for 33.3% (*n* = 13) and pregnancy for 2.6% (*n* = 1). Results are summarized in Table 3.

**Table 3:** Causes of TKI discontinuation according to transcript type (e13a2 versus e14a2) (n = 39)

Causes of TKI discontinuation	*N* = (%)		
	e14a2	e13a2	*p* =
Failure	7 (30.4)	4 (25)	
Side-effect	6 (26.1)	8 (50)	
TFR-attempt	10 (43.5)	3 (18.8)	
Other	0 (0)	1 (6.2)	
Total	23 (100)	16 (100)	.19

Abbreviations: TFR, treatment-free remission; TKI, tyrosine kinase inhibitor. Results were compared using Fischer’s exact method.

### MMR and MR^4.5^: univariate and multivariate analysis

We then performed univariate and multivariate analysis to examine the role of the transcript’s type on achieving molecular responses.

In the multivariate model, a high platelet count of over 300 G/L was the only factor significantly predicting better MMR rates (Supplementary Table 1). Considering MR^4.5^, our multivariate model showed that expressing e14a2 transcript was predictive of MR^4.5^ among other variables: sex (male), high WBC count and high platelet count. These results are summarized in Table 4.

**Table 4:** Multivariate analysis to identify factors predictive of MR^4.5^

*Univariate analysis*	OR	[95% CI OR]	MR^4.5^ N (%)	No MR^4.5^ N (%)	*p* =
**Male**					
Yes	0.19	[0.09–0.41]	20 (33.3)	47 (72.3)	< .001
No			40 (66.7)	18 (27.7)	
**Splenomegaly**					
Yes	0.29	[0.11–0.79]	6 (10)	18 (27.7)	.02
No			54 (90)	47 (72.3)	
**WBC count (> 150 × 10^9^/L)**					
Yes	0.22	[0.10–0.49]	13 (21.7)	36 (55.4)	< .001
No			47 (78.3)	29 (44.6)	
**PLT count (> 300 G/L)**					
Yes	5.20	[2.29–11.75]	49 (81.7)	30 (46.2)	< .001
No			11 (18.3)	35 (53.8)	
**Hemoglobin (> 120 g/L)**					
Yes	1.50	[0.73–3.05]	29 (48.3)	25 (38.5)	.27
No			31 (51.7)	40 (61.5)	
**High ELTS score**					
Yes	0.40	[0.13–1.21]	5 (8.3)	12 (18.5)	.11
No			55 (91.7)	53 (81.5)	
**High Sokal score**					
Yes	0.71	[0.30–2.00]	12 (20)	17 (26.1)	.42
No			48 (80)	48 (73.9)	
**ACA or variant translocation**					
Yes	0.73	[0.27–1.95]	8 (13.3)	11 (17.5)	.53
No			52 (86.7)	52 (82.5)	
**Transcript e14a2, alone or co-expressed**					
Yes	3.21	[1.51–6.80]	44 (73.3)	30 (46.2)	.002
No			16 (26.7)	35 (53.8)	

*Multivariate analysis*	OR	[95% CI OR]	*p* =
**Male**	0.28	[0.11–0.71]	.007
**Splenomegaly**	0.56	[0.13–2.46]	.45
**WBC count**	0.29	[0.10–0.84]	.02
**Platelets**	3.68	[1.39–9.76]	.009
**ELTS score**	2.42	[0.43–13.38]	.31
**e14a2**	3.25	[1.30–8.18]	.01

Abbreviations: ACA, additional chromosome abnormalities in Ph+ cells; EUTOS, European Treatment and Outcome Study; ELTS; PLT, platelets; WBC, white blood cells. Variables with *p* values inferior to 0.20 in univariate analysis were included in the multivariate model.

## DISCUSSION

A majority of patients with CP-CML expressed e14a2 or e13a2 *BCR-ABL1* transcript. The influence of a transcript type on outcome or molecular response has been questioned for a long time. In the Imatinib era, numerous studies suggested better and faster responses in patients expressing e14a2 transcript as discussed in a recent review by Ercaliskan et al. [21].

As few data evaluating the impact of transcript types in patients treated by second tyrosine kinase inhibitors were available, we retrospectively evaluated in this study the influence of transcript type in 114 patients with CP-CML treated frontline with Nilotinib. Patients expressing e14a2 transcripts had deeper and faster molecular responses, when considering MMR and MR^4.5^, compared to those expressing e13a2, despite the use of second-generation TKI. These results are consistent with data reported by Jain et al. [22]. Similarly, out of 237 patients, Yi-Jiun Su et al. [23] found that e14a2 patients had a better rate of MMR at 12 months compared to e13a2 patients despite no difference in median time to MMR, median time to MR^4.5^ or in the cumulative incidence of MR^4.5^. A preliminary study from the GIMENA CML WP [24] suggested that patients expressing e13a2 had a trend to a lower cumulative incidence of MMR and MR^4.0^ but these differences were not significant. However, these results became significant when grouping together patients expressing e14a2 and patient co-expressing both transcripts.

One explanation to our findings could be linked to the differential amplification between the two transcripts. As suggested by Hanfstein et al. [10], we speculated that a better amplification of the smaller transcript (*i. e.* e13a2) could result in a majored and biased quantification. Indeed, the European Against Cancer (EAC) protocol used a common forward primer in *BCR* exon 13, resulting in a 76 bp difference in amplification between the 2 transcripts. Therefore, we first evaluated PCR amplification efficiencies and found no significant difference between e14a2 and e13a2 transcripts, allowing the direct comparison of quantification values whatever the transcript type.

When considering the cumulative probability of achieving MR^4.5^, it is interesting to see that the clearance profile of *BCR-ABL1* is very similar in the first 18 months between the two transcript subtypes. This exponential biphasic declination pattern had already been identified with imatinib [25] and confirmed independently of the TKI given frontline [26]. The authors explained this phenomenon by the elimination of differentiated leukemia cells first and then the leukemia progenitors.

Some data are attractive trails to explain the differential clearance of both types of transcripts. Interestingly, Lucas et al. [12] evaluated the pCrKL/CrKL ratio, a surrogate of BCR-ABL1 kinase activity [27], and found that patients expressing e13a2 had a higher tyrosine kinase activity. The spatial structure of the 2 proteins seems to be different, as illustrated by Hai et al. [28], and could impact the interaction with kinase inhibitors. More recently, Pagani et al. [29] described, even in a small number of patients, a lower *BCR-ABL1* mRNA/*BCR-ABL1* DNA ratio in e13a2 versus e14a2 patients, consistent with an experimental low-*BCR-ABL1* murine model showing a reduced imatinib sensitivity. This low *BCR-ABL1* expression in e13a2 patients could lead to incorrect classification for treatment decisions, including MR^4.5^ or MR^5^ evaluations that guide TKI discontinuation.

The role of the additional 25 amino-acids (coded by the e14 exons) between e14a2 and e13a2 transcripts has also been questioned. Clark et al. first demonstrated a specific immunologic role of e14a2 peptide compared to e13a2 and its potential to induce cytotoxic T-lymphocyte response [30]. This Immunologic role could also have an impact on TFR.

Our study includes several limitations. Molecular evaluation was not performed centrally. However, the techniques used are similar and there is a national and international consensus on their implementation [31]; all the results are aligned on the international scale by the 5 laboratories involved in our study. Given the effectiveness of current treatments and especially with second-generation inhibitors, it is necessary to have a sufficient number of patients and in this context, our retrospective cohort on a limited number of patients have to be confirmed in a larger one, as what had been done with imatinib.

We only focused on nilotinib, the only second generation TKI approved and reimbursed in France. It may be interesting to evaluate the impact of other drugs approved as first line therapies by the FDA or the EMA such as dasatinib or bosutinib. Finally, the mechanism underlying the different response rates to treatment among molecular subgroups need to be better understood.

Overall, we conclude that even treated with nilotinib first line, patients with CP-CML expressing *BCR-ABL1* e13a2 transcript seems to have a lower rate of MMR and MR^4.5^.

## MATERIALS AND METHODS

### Patients

This is a retrospective and multicentric study. All patients with newly diagnosed CP-CML and treated frontline with nilotinib in Annecy, Clermont-Ferrand, Lyon, Saint-Etienne and Toulouse hospitals were enrolled. The patients were diagnosed between 2007 and 2017 and treated inside or outside clinical trials. Patients expressing e14a2 and e13a2 transcripts were analyzed. Patients with atypical *BCR-ABL1 and* co-expressors transcripts or treated in combination with interferon were excluded from this analysis. Demographic and disease characteristics were assessed at baseline. Written information has been given to all participants and a non-opposition statement was obtained according to national and institutional requirements.

### Response criteria

Response criteria were based on the 2013 European Leukemia Net (ELN) recommendations for the management of CML [32]. Complete hematological response (CHR) was defined by platelets < 450 G/L, white cells < 10 G/L, no circulating immature myeloid cells, < 5% basophils on differential and no palpable splenomegaly. Complete cytogenetic response (CCyR) was defined by no Ph^1^ cells in karyotype. Early molecular response (EMR) was characterized by a *BCR-ABL1*^IS^ ≤ 10% at 3 months. Major molecular response (MMR) was obtained when *BCR-ABL1*^IS^ was lower or equal to 0.10% and MR^4.5^ with a BCR-ABL1^IS^ < 0.0032%.

All molecular classifications were based on *BCR-ABL1* control ratios and standardized according to the International Scale or IS [33]. Follow-up was similar in all five centers with at least real-time quantitative PCR performed at 3, 6, 12 and 18 months and cytogenetic analysis at 6 months and until CCyR. Event free survival (EFS) was measured from the initiation of treatment to the date of any of the following events while on therapy: loss of CHR, loss of CCyR, resistance, progression to accelerated or blastic phase, stopping nilotinib for toxicity or death from any cause. Overall survival (OS) was defined from the time of treatment initiation to the date of death from any cause at any time or date of last follow-up.

### Transcripts typing

RNA extraction from Ficoll-separated peripheral blood cells and reverse transcription were performed at the time of diagnosis for the majority of patients or retrospectively from a sample collected at the diagnosis when the results were not available. PCR were performed using Veriti thermal cycler (Applied Biosystems, Carlsbad, CA, USA).

The type of *BCR-ABL1* transcript was identified according to the size of RT–PCR product as previously described [34].

### Transcripts quantification

Both Transcripts were quantified in blood samples using EAC protocol [35] MMLV-RT was changed for a Superscript VILO-RT (Life TechnologiesThermo Fischer Sicentific) to ensure a minimum of 32000 copies of *ABL1* control gene to define at least MR^4.5^ for each sample. All results are expressed and standardized according to IS.

In order to investigate a possible bias in amplification according to transcript type, RQ-PCR efficiencies for e13a2 and e14a2 were compared, based on the slope of the standard curves resulting from serial dilutions of cDNA extracted from K562 (e14a2) and BV173 (e13a2) cell lines. A total of 48 standard curves (24 for e13a2 and 24 for e14a2) in 4 different experiments were performed. Furthermore, to demonstrate the ability of the EAC assay to accurately determine the number of longer transcripts produced by an e14a2 fusion RNA, we designed a specific assay for e14a2 transcript (Forward Primer: ATGGGTTTCTGAATGTCATCG) and compared the PCR efficiencies between EAC protocol and specific e14a2 protocol in a e14a2 cell line (K562). A total of 10 standard curves in two different experiments were performed.

### Statistical analysis

PCR efficiencies were compared using the Wilcoxon-Mann-Whitney test. Baseline characteristics of patients according to the type of transcripts were performed using Pearson’s chi square test, Kruskal-Wallis test or one way-ANOVA, as appropriate.

The cumulative incidence of MMR, MR^4.5^, EFS and OS was estimated using the Kaplan–Meier method. Patients who discontinued treatment whatever the cause were censored at the date of discontinuation. Differences were compared with the log-rank test.

Univariate and multivariate analyses were performed to identify whether the subtype of transcript could predict the molecular responses. The unadjusted significance level of 0.05 was applied to all statistical tests.

Statistical analyses were carried out using STATA/SE version 14.1 (Stata corp., College Station, TX, USA).

## SUPPLEMENTARY MATERIALS



## Abbreviations

ACA: Additional Clonal Abnormality
ALL: Acute Lymphoblastic Leukemia
CCyR: Complete Cytogenetic Response
CHR: Complete Hematologic Response
DNA: DeoxyriboNucleic acid
ELN: European leukemia Net
ELTS: Eutos Long Terme Survival
EUTOS: EUropean Treatment Outcome Study
MR: Molecular response
MMR: Major molecular response
PCR: polymerase Chain reaction
RNA: RiboNucleic acid
RT-PCR: Reverse Transcriptase – Polymerase Chain reaction
TKI: Tyrosine Kinase Inhibitors
TFR: Treatment free remission
WBC: White Blood Count
