# Supplementary material for: Influence of major BCR-ABL1 transcript subtype on outcome in patients with chronic myeloid leukemia in chronic phase treated frontline with nilotinib
Source: Oncotarget. 2020 Jun 30;11(26):2560–70. doi: 10.18632/oncotarget.27652 (PMC7335668; doi:10.18632/oncotarget.27652)
Supplement: Supplementary file 1 [file oncotarget-11-2560-s001.pdf]

# Influence of major *BCR-ABL1* transcript subtype on outcome in patients with chronic myeloid leukemia in chronic phase treated frontline with nilotinib

## SUPPLEMENTARY MATERIALS

### Supplementary Table 1: Multivariate analysis to identify factors predictive of $MR^{4.0}$

| *Univariate analysis* | OR | [95% CI OR] | MMR at 12 months N (%) | No MMR at 12 months N (%) | *p* = |
|---|---|---|---|---|---|
| **Male** | | | | | |
| Yes | 0.49 | [0.24–1.00] | 31 (45.6) | 36 (63.2) | .05 |
| No | | | 37 (54.6) | 21 (36.8) | |
| **Splenomegaly** | | | | | |
| Yes | 0.21 | [0.08–0.57] | 6 (8.8) | 18 (31.6) | .002 |
| No | | | 62 (91.2) | 39 (68.4) | |
| **WBC count (> 150 × $10^3$/µL)** | | | | | |
| Yes | 0.30 | [0.14–0.64] | 18 (26.5) | 31 (54.4) | .002 |
| No | | | 50 (73.5) | 26 (45.6) | |
| **PLT count (> 300 × $10^3$/µL)** | | | | | |
| Yes | 2.68 | [1.27–5.67] | 50 (73.5) | 29 (50.9) | .009 |
| No | | | 18 (26.5) | 28 (49.1) | |
| **Hemoglobin (> 120 g/L)** | | | | | |
| Yes | 2.44 | [1.17–5.08] | 36 (52.9) | 18 (31.6) | .02 |
| No | | | 31 (47.1) | 39 (68.4) | |
| **High ELTS score** | | | | | |
| Yes | 0.30 | [0.10–0.90] | 5 (7.4) | 12 (21.1) | .03 |
| No | | | 63 (92.6) | 45 (78.9) | |
| **High Sokal score** | | | | | |
| Yes | 0.87 | [0.38–2.00] | 29 (23.2) | 14 (24.6) | .74 |
| No | | | 96 (76.8) | 43 (75.4) | |
| **ACA or variant translocation** | | | | | |
| Yes | 2.01 | [0.71–5.68] | 13 (19.4) | 6 (10.7) | .18 |
| No | | | 54 (80.6) | 50 (89.3) | |
| **Transcript e14a2, alone or co-expressed** | | | | | |
| Yes | 2.17 | [1.05–4.48] | 46 (67.6) | 28 (49.1) | .04 |
| No | | | 22 (32.4) | 29 (50.9) | |

| *Multivariate analysis* | OR | [95% CI OR] | *p* = |
|---|---|---|---|
| **Male** | 0.74 | [0.31–1.81] | .51 |
| **Splenomegaly** | 0.27 | [0.07–1.06] | .06 |
| **WBC count** | 0.58 | [0.22–1.54] | .27 |
| **Platelets** | 2.58 | [1.03–6.47] | .04 |
| **Hemoglobin** | 1.84 | [0.76–4.47] | .18 |
| **ACA or variant translocation** | 3.04 | [0.91–10.10] | .07 |
| **ELTS score** | 1.81 | [0.38–8.53] | .45 |
| **e14a2** | 2.15 | [0.93–4.97] | .07 |

Abbreviations: OR, odds ratio; ACA, additional chromosome abnormalities in Ph+ cells; EUTOS, European Treatment and Outcome Study; ELTS; PLT, platelets; WBC, white blood cells. Variables with *p* values inferior to 0.20 in univariate analysis were included in the multivariate model.
